# A suppressive role of guanine nucleotide-binding protein subunit beta-4 inhibited by DNA methylation in the growth of anti-estrogen resistant breast cancer cells

**DOI:** 10.1186/s12885-018-4711-0

**Published:** 2018-08-13

**Authors:** Bo Wang, Dongping Li, Rocio Rodriguez-Juarez, Allison Farfus, Quinn Storozynsky, Megan Malach, Emily Carpenter, Jody Filkowski, Anne E. Lykkesfeldt, Olga Kovalchuk

**Affiliations:** 1 Department of Biological Sciences, University of Lethbridge, Lethbridge, AB Canada; 2 Department of Biochemistry, Qiqihar Medical University, Qiqihar, People’s Republic of China; 3 Breast Cancer Group, Cell Death and Metabolism, Danish Cancer Society Research Center, Strandboulevarden, Copenhagen, Denmark; 4 Hepler Hall, University of Lethbridge, 4401 University Drive, Lethbridge, AB T1K 3M4 Canada

**Keywords:** Antiestrogen resistance, Breast cancer, DNA methylation, Fulvestrant, GNB4, Tamoxifen

## Abstract

**Background:**

Breast cancer is the most common malignancy in women worldwide. Although the endocrine therapy that targets estrogen receptor α (ERα) signaling has been well established as an effective adjuvant treatment for patients with ERα-positive breast cancers, long-term exposure may eventually lead to the development of acquired resistance to the anti-estrogen drugs, such as fulvestrant and tamoxifen. A better understanding of the mechanisms underlying antiestrogen resistance and identification of the key molecules involved may help in overcoming antiestrogen resistance in breast cancer.

**Methods:**

The whole-genome gene expression and DNA methylation profilings were performed using fulvestrant-resistant cell line 182^R^-6 and tamoxifen-resistant cell line TAM^R^-1 as a model system. In addition, qRT-PCR and Western blot analysis were performed to determine the levels of mRNA and protein molecules. MTT, apoptosis and cell cycle analyses were performed to examine the effect of either guanine nucleotide-binding protein beta-4 (GNB4) overexpression or knockdown on cell proliferation, apoptosis and cell cycle.

**Results:**

Among 9 candidate genes, GNB4 was identified and validated by qRT-PCR as a potential target silenced by DNA methylation via DNA methyltransferase 3B (DNMT3B). We generated stable 182^R^-6 and TAM^R^-1 cell lines that are constantly expressing GNB4 and determined the effect of the ectopic GNB4 on cell proliferation, cell cycle, and apoptosis of the antiestrogen-resistant cells in response to either fulvestrant or tamoxifen. Ectopic expression of GNB4 in two antiestrogen resistant cell lines significantly promoted cell growth and shortened cell cycle in the presence of either fulvestrant or tamoxifen. The ectopic GNB4 induced apoptosis in 182^R^-6 cells, whereas it inhibited apoptosis in TAM^R^-1 cells. Many regulators controlling cell cycle and apoptosis were aberrantly expressed in two resistant cell lines in response to the enforced GNB4 expression, which may contribute to GNB4-mediated biologic and/or pathologic processes. Furthermore, knockdown of GNB4 decreased growth of both antiestrogen resistant and sensitive breast cancer cells.

**Conclusion:**

GNB4 is important for growth of breast cancer cells and a potential target for treatment.

**Electronic supplementary material:**

The online version of this article (10.1186/s12885-018-4711-0) contains supplementary material, which is available to authorized users.

## Background

Breast cancer is the most common malignancy found in women worldwide and the second leading cause of cancer-related deaths among North American women [1]. Globally, it is estimated that 1.67 million new cases were diagnosed, and 522,000 women died from this disease in 2012 (GLOBOCAN 2012). Approximately 60% of these deaths were contributed by less developed countries. Although the exact etiology of breast cancer is currently unknown, the estrogen/estrogen-receptor (ER) signaling may play a crucial role in the development of this disease [2–5]. Furthermore, sustained estrogenic exposure may also increase the risk of breast, ovarian, and endometrial cancers [6].

Estrogen receptor alpha (ERα) and beta (ERβ), two ligand-inducible transcription factors, are members of the steroid/thyroid receptor superfamily that primarily mediate estrogen’s biological function through binding [7]. ERα and ERβ are encoded by either *ESR1* or *ESR2* genes that are composed of 595 and 530 amino acids, respectively. Both eventually form an N-terminal domain (NTD), a DNA-binding domain (DBD), and a ligand-binding domain (LBD) [8]. Sequence analysis indicates that ERα and ERβ display ~ 97% similarity in the DBD and 59% in the LBD, whereas they display only 16% similarity in the NTD [8]. This implicates a functional similarity and difference between these two ERs. For instance, once activated via estrogen binding, both dimerized ERs can either bind to the estrogen-response element (ERE) in the DNA or interplay with other transcription factors, such as AP1, Sp1, and NF-κB [9], eventually influencing the transcription of genes. However, ERα may predominantly bind to ERE elements [10], while ERβ may primarily interact with AP1 sites [11]. Furthermore, as demonstrated, ERα is a key player in promoting cell growth and proliferation [12, 13], whereas ERβ plays an important role in anti-proliferation, differentiation, and apoptosis in human malignancies, including breast cancer [14, 15].

Because ERα is expressed in 70% of breast cancers [16], and the proliferation of these ERα-positive breast cancers is largely dependent on estrogen/ERα signaling [17], the endocrine therapy that targets estrogen/ERα signaling has been well established as an effective adjuvant treatment for patients with ERα-positive breast cancers [18]. The endocrine-therapy agents that are currently used for ERα-positive breast cancer include fulvestrant (also known as ICI 182,780 and faslodex, the ER downregulator that selectively downregulates and/or degrades ERα), tamoxifen (the ER modulator that selectively antagonizes ERα function), and aromatase inhibitors (e.g. letrozole and anastrozole, which inhibit estrogen production by attenuating aromatase activity) [17, 19]. As an important adjuvant therapy, continuing 10-year tamoxifen treatment, when compared with 5-year exposure, has been shown to further reduce the risk of disease recurrence and mortality in a randomized trial of women with ER-positive breast cancers [20]. Unfortunately, long-term exposure may eventually lead to the development of acquired resistance to these drugs [21–23], which is a serious clinical problem in hormonal therapy. However, the underlying mechanisms are not completely understood.

In this study, we globally analyzed genomic DNA methylation, correlated with gene expression profiling, and identified *GNB4* that was silenced by DNMT3B-mediated DNA methylation in both fulvestrant-resistant (MCF-7/182^R^-6) and tamoxifen-resistant (MCF-7/TAM^R^-1) breast cancer cell lines. Ectopic expression of GNB4 enhanced proliferation of MCF-7/182^R^-6 and MCF-7/TAM^R^-1 cell lines in response to either fulvestrant or tamoxifen, while it shortened G2 and S phases in the cell cycle. We also noted that the ectopic expression of GNB4 induced apoptosis in the MCF-7/182^R^-6 cell line, whereas it attenuated the induction of apoptosis in the MCF-7/TAM^R^-1 cell line. Cell-cycle and apoptosis regulators were aberrantly expressed in these cell lines in response to the ectopic GNB4 expression. In contrast, siRNA-mediated knockdown of GNB4 inhibited proliferation of two resistant cell lines in the presence of either fulvestrant or tamoxifen, and induced either S phase arrest or apoptosis. Our results provide novel insight into the role of GNB4 in the growth of both antiestrogen-resistant and sensitive breast cancer cells and may represent a target for treatment of breast cancer.

## Methods

### Cell culture

The MCF-7/S0.5 (S05), MCF-7/182^R^-6 (182^R^-6), and MCF-7/TAM^R^-1 (TAM^R^-1) cell sublines were developed by Dr. Anne Lykkesfeldt (Breast Cancer Group, Cell Death and Metabolism, Danish Cancer Society Research Center, DK-2100, Copenhagen, Denmark). ICI 182,780 (Faslodex, fulvestrant) and tamoxifen-resistant sublines, 182^R^-6 and TAM^R^-1, respectively, are derived from S05 as described elsewhere [24, 25]. These cell lines were cultured in a DMEM/F-12 medium with 2.5 mM L-Glutamine, without HEPES and phenol red (HyClone), and supplemented with 1% heat-inactivated fetal bovine serum (HyClone). Additionally, for 182^R^-6 and TAM^R^-1 sublines were regularly supplemented with 0.1 μM ICI 182,780 and 1 μM tamoxifen, respectively. Human mammary epithelial cells (HMEC) purchased from ThermoFisher Scientific (Cat# A10565) were cultured in a HuMEC basal serum-free medium (ThermoFisher Scientific) containing HuMEC supplement (ThermoFisher Scientific), 100 IU/mL penicillin, and 100 mg/mL streptomycin. All cell lines were incubated at 37 °C in a humidified atmosphere of 5% CO_2_.

## Whole-genome gene expression profiling

Total RNA was isolated from S05, 182^R^-6, and TAM^R^-1 cells using an Illustra RNAspin mini kit according to the manufacturer’s instructions (GE Healthcare Life Sciences). Quantification, purity, and integrity of the RNA samples were measured using a NanoDrop 2000c spectrophotometer (Thermo Scientific) and an Agilent 2100 bioanalyzer (Santa Clara). RNA samples with RIN values of seven or higher were used for further analysis. Procedures for library preparation, hybridization, detection, BeadChip statistical analysis, and data processing have been described previously [19]. Heatmaps were generated by Dr. Yaroslav Ilnytskyy for genes that were differentially expressed between any of the groups (ANOVA type analysis with p.adjusted < 0.001) and for top 1000 most variable probes in DNA methylation.

### Whole-genome DNA methylation profiling

DNA was extracted from cells using the DNeasy Blood and Tissue Kit (QIAGEN) and treated with DNase-free RNase (Sigma) according to the manufacturer’s protocols. The collected DNA was bisulfite converted using the EZ DNA Methylation Kit (Zymo Research) according to the manufacturer’s protocols. Methylation was measured using the Infinium assay on the Illumina platform. Data was collected from the > 27,000 probes represented on the HM27 microarray. These probes contain CpG dinucleotides from selected loci throughout the genome. All steps were carried out according to the manufacturer’s specifications and with Illumina-supplied reagents. Briefly, bisulfate-converted samples were amplified overnight, fragmented, and purified. The re-suspended samples were hybridized overnight to the microarray, which harboured millions of bead-bound 50-mer oligos. Each interrogated loci is represented by two bead types: a methylated type (“C” remains a “C”) and an unmethylated type (“C” become a “T”). Hybridized chips were washed to remove unbound and/or non-specific DNA fragments. The resulting oligo-sample hybrid was then extracted with a biotin-linked dideoxy cytosine and stained with streptavidin. The relative intensity of the unmethylated bead to the methylated bead for each allele provides a measure of relative methylation levels.

### Quantitative real-time RT-PCR (qRT-PCR)

Total RNA, isolated from the indicated cell lines with TRIzol reagent (Invitrogen), was subjected to qRT-PCR using an iScript™ Select cDNA Synthesis kit and an SsoFast^MT^ EvaGreen Supermix (Bio-Rad) with the primer set specifically to GNB4 (PrimePCR SYBR Green Assay, Bio-Rad) according to the manufacturer’s instructions. Glyceraldehyde-3-phosphate dehydrogenase gene (*GAPDH*) was used as the internal control to standardize and test the RNA integrity with a sequence for the forward primer, 5′-GAA GGC TGG GGC TCA TTT-3′, and for the reverse primer, 5’-CAG GAG GCA TTG CTG ATG AT-3′ [26]. All qRT-PCR experiments were performed in triplicate, the data was analyzed using the comparative Ct method, and the results are shown as a fold induction of mRNA.

### Knockdown of DNMT3B

182^R^-6 and TAM^R^-1 cells grown to 80% confluency were transiently transfected with either 200 nM Dnmt3b siRNA (Santa Cruz Biotechnologies) or 200 nM AllStars Negative Control siRNA (QIAGEN) using Lipofectamine 3000 (Invitrogen) according to the manufacturer’s instructions. Seventy-two hours after transfection, the total RNA was isolated and subjected to qRT-PCR analysis using a primer set specifically to GNB4 (Bio-Rad) according to the manufacturer’s instructions, and the whole cellular lysates were prepared and subjected to Western blot analysis using antibodies against DNMT3B and GNB4.

### Generation of GNB4 expression construct and GNB4 stable-expression cell lines

The coding sequence of GNB4 was amplified by RT-PCR using total RNA isolated from HMEC. The PCR product was then cloned into a pGEM-T easy vector (Promega), released by digestion with *Eco*R I and *Bam*H I, and subcloned into a pEGFP-C1 vector (CloneTech) to generate pEGFP-GNB4. Sequence identity was confirmed by automatic sequencing. Primers used here for amplifying the GNB4 coding sequence are as follows: GNB4-F (5′-GAG AAT TCT ATG AGC GAA CTG GAA C-3′) and GNB4-R (5′-GGG GAT CCA TTC CAG ATT CTA AG-3′).

182^R^-6 and TAM^R^-1 cells grown to 80% confluency were transfected with either pEGFP-GNB4 or pEGFP-C1 using Lipofectamine 3000 (Invitrogen). 24 h after transfection, G418 was added to final concentrations of 800 μg/mL and 400 μg/mL for 182^R^-6 and TAM^R^-1 cell lines, respectively, to kill the negative cells. The positive cells stably expressing either GFP or GFP-GNB4 were further selected with cell sorting (University of Calgary).

### MTT assay

The MTT assay was performed as described previously [27]. Briefly, 3.0 × 10^3^ 182^R^-6 or TAM^R^-1 or S05 cells transiently transfected with either 30 nM GNB4 (Gβ4) siRNA (Santa Cruz Biotechnology) or 30 nM AllStars negative control siRNA (QIAGEN), or stably expressing either GFP or GFP-GNB4 were plated in 96-well plates. The 3-(4,5-Dimethylthiazol-2-yl)-2,5-diphenyl tetrazolium bromide (MTT) assays were carried out using a Cell Proliferation Kit I (Roche Diagnostics GmbH) according to the manufacturer’s instructions. The spectrophotometric absorbance of samples was measured at 595 nm using a microtiter plate reader (FLUOstar Omega).

### Cell cycle and apoptosis analyses

182^R^-6 or TAM^R^-1 cells stably expressing either GFP or GFP-GNB4 grown to 90% confluency were harvested for cell-cycle and apoptosis analyses that were performed with a BD FACSCanto™ II Flow Cytometer (BD Biosciences) using a GFP-Certified Nuclear-ID Red Cell Cycle Analysis Kit (Enzo) and an Annexin V-Cy3 Apoptosis Kit Plus (BioVision) according to the manufacturer’s instructions.

182^R^-6 or TAM^R^-1 cells transiently transfected with either 30 nM GNB4 siRNA (Santa Cruz Biotechnology) or 30 nM AllStars negative control siRNA (QIAGEN), 72 h (for TAM^R^-1 line) or 96 h (for 182^R^-6 line) after transfection, the cells were harvested for cell-cycle and apoptosis analyses that were performed with a BD FACSCanto™ II Flow Cytometer (BD Biosciences) using propidium iodide staining solution and FITC Annexin V Apoptosis Detection kit II (BD Biosciences) according to the manufacturer’s instructions.

### Western blot analysis

The indicated cells grown to 90% confluency were rinsed twice with ice-cold PBS and scraped off the plate in a radioimmunoprecipitation assay buffer (RIPA). We electrophoresed 30–100 μg of protein per sample on 6% or 10% SDS-PAGE and electrophoretically transferred to a PVDF membrane (Amersham Hybond™-P, GE Healthcare) at 4 °C for 1.5 h. Blots were incubated for one hour with 5% nonfat dry milk to block nonspecific binding sites and then incubated with polyclonal/monoclonal antibodies against BAX (BCL2-associated X protein), BCL2 (B-cell CLL/Lymphoma 2), DNMT3A (DNA methyltransferase 3A), GNB4, pAKT1/2/3 (phosphorylated AKT1/2/3) (Santa Cruz Biotechnology) or AKT1 (v-AKT murine thymoma viral oncogene homolog 1), DNMT1 (DNA methyltransferase 1), DNMT3B, p21^(Waf1/Cip1)^ (Abcam) or CDK2 (cyclin-dependent kinase 2), CDK6 (cyclin-dependent kinase 6), cyclin A2, cyclin D1, cyclin E1, ERK1/2 (extracellular signal-regulated kinase 1/2), MeCP2 (methyl-CpG-binding protein 2), and pERK1/2 (phosphorylated ERK1/2) (Cell Signaling Technology) at 4 °C overnight. Immunoreactivity was detected using a peroxidase-conjugated antibody and visualized by an ECL Plus Western Blotting Detection System (GE Healthcare). The blots were stripped before re-probing with antibody against actin (Santa Cruz Biotechnology).

### Statistical analysis

The student’s *t*-test was used to determine the statistical significance between groups in GNB4 expression, cell growth, cell cycle, and apoptosis. *p* < 0.05 was considered significant.

## Results

### Epigenetic silencing of *GNB4* via DNMT3B-mediated DNA methylation

**Fig. 1:**
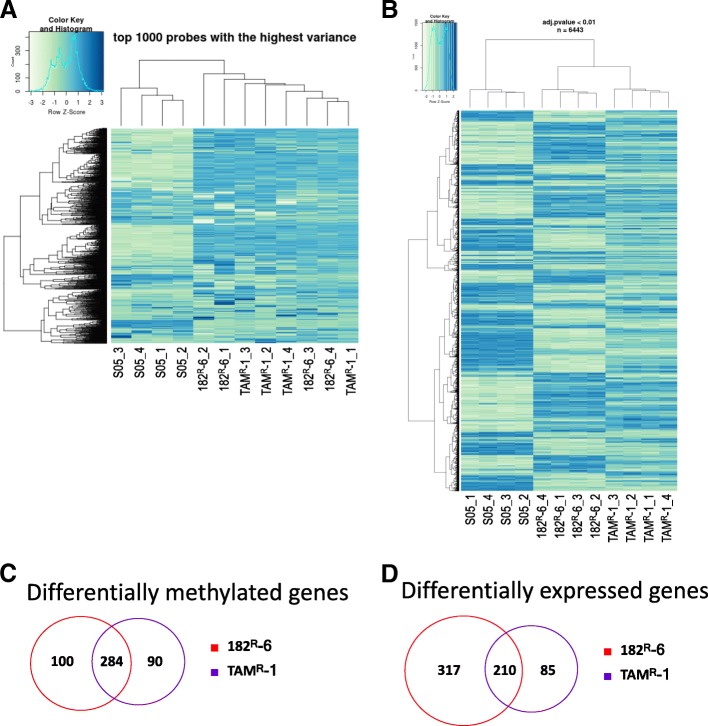
Whole-genome DNA methylation and gene expression analyses. **a**, Heatmap of differentially methylated genes. DNA extracted from S05, 182^R^-6, and TAM^R^-1 cells was treated with DNase-free RNase and bisulfite converted; DNA methylation assay, data collection, and analysis were performed as described in “Methods”. **b**, Heatmap of differentially expressed genes. Total RNA isolated from S05, 182^R^-6, and TAM^R^-1 cells was subjected to gene expression profiling; the detailed procedures for library preparation, hybridization, detection, BeadChip statistical analysis, and data processing have been described previously [19]. **c** and **d**, The number of differentially methylated genes (**c**) and differentially expressed genes (**d**) was presented with a Venn diagram

**Fig. 2:**
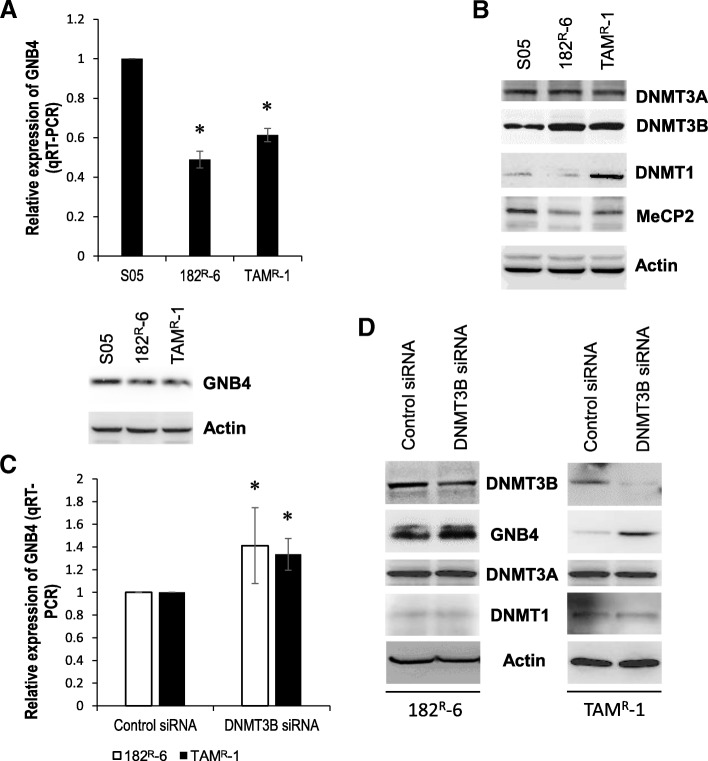
Silencing of GNB4 in 182^R^-6 and TAM^R^-1 cells via DNMT3B. **a**, Total RNA isolated from S05, 182^R^-6, and TAM^R^-1 cells was subjected to qRT-PCR using a primer set specific to GNB4. Whole cellular lysate was prepared from S05, 182^R^-6, and TAM^R^-1 cells, and Western blot analysis was performed using an antibody against GNB4. **b**, Whole cellular lysate was prepared from S05, 182^R^-6, and TAM^R^-1 cells, and Western blot analysis was performed using antibodies against DNMT1, DNMT3A, DNMT3B, and MeCP2. **c**, 182^R^-6 and TAM^R^-1 cells were transiently transfected with either 200 nM DNMT3B siRNA or 200 nM negative control siRNA; 72 h after transfection, total RNA isolated from these cells was subjected to qRT-PCR using a primer set specific to GNB4. **d**, Seventy-two hours after transfection, whole cellular lysate prepared from 182^R^-6 and TAM^R^-1 cells was transfected with either 200 nM DNMT3B siRNA or 200 nM negative control siRNA, and was subjected to Western blot analysis using antibodies against DNMT1, DNMT3A, DNMT3B and GNB4. Asterisk indicates *p* < 0.03

To explore the contribution of DNA methylation to the development of the acquired resistance to endocrine therapy in breast cancer, using a fulvestrant-resistant 182^R^-6 cell line, a tamoxifen-resistant TAM^R^-1 cell line, and their parental line S05 as a model system, we performed whole-genome DNA methylation and gene-expression profilings. We identified 284 genes as common targets of DNA methylation in both 182^R^-6 and TAM^R^-1 cell lines (Fig. 1a and c). Differential expression in both antiestrogen-resistant cell lines was evident in 210 genes (Fig. 1b and d). We then correlated the expression of 210 genes with their DNA methylation status and identified nine downregulated genes, including *annexin A6* (*ANXA6*), *dual-specificity phosphatase 2* (*DUSP2*), *ephrin B3* (*EFNB3*), *guanine nucleotide-binding protein beta-4* (*GNB4*), *methyltransferase-like 7A* (*METTL7A*), *p8* (also known as *candidate of metastasis 1*, *COM1*), *protease serine 23* (*PRSS23*), *S100 calcium-binding protein A4* (*S100A4*), and *tripartite motif-containing 4* (*TRIM4*). Their promoters were hypermethylated in both 182^R^-6 and TAM^R^-1 cell lines (Table 1), while only GNB4 was validated to be downregulated in both cell lines by qRT-PCR and Western blot analyses (Fig. 2a). Interestingly, Western blot analysis showed that DNMT3B was upregulated, while MeCP2 was downregulated in both cell lines. This implicates a common role of DNMT3B in both cell lines in GNB4 promoter hypermethylation, although DNMT1 may also play a role in the TAM^R^-1 cell line (Fig. 2b). To test our hypothesis, DNMT3B was transiently knocked down using siRNA. Seventy-two hours after transfection, DNMT3B was downregulated by siRNA; as a result, GNB4 was upregulated at both mRNA and protein levels in 182^R^-6 and TAM^R^-1 cell lines (Fig. 2c and d); whereas, DNMT3B siRNA had no effect on the expression of both DNMT1 and DNMT3A (Fig. 2d). Our results suggest that GNB4 was epigenetically silenced in 182^R^-6 and TAM^R^-1 cell lines by DNA methylation via DNMT3B.

**Table 1:** Genes downregulated and their promoters hypermethylated

182^R^-6			TAM^R^-1		
Symbol	Expression	Methylation	Symbol	Expression	Methylation
ANXA6	-1.84973	1.832057	ANXA6	-1.67854	1.915985
DUSP2	-1.56577	2.003189	DUSP2	-1.52014	2.115186
EFNB3	-2.55586	1.300826	EFNB3	-1.73551	1.349243
GNB4	-1.50833	1.418815	GNB4	-1.26312	1.129974
METTL7A	-1.42021	1.223772	METTL7A	-1.01184	1.473379
P8	-1.55328	1.688645	P8	-1.26429	1.672422
PRSS23	-1.17273	1.018398	PRSS23	-2.00926	1.17323
S100A4	-1.8409	1.529188	S100A4	-2.17128	1.478892
TRIM4	-1.57438	1.767231	TRIM4	-1.08051	1.945837

### Ectopic expression of GNB4 enhanced proliferation of antiestrogen-resistant breast cancer cells in the presence of antiestrogen drugs

**Fig. 3:**
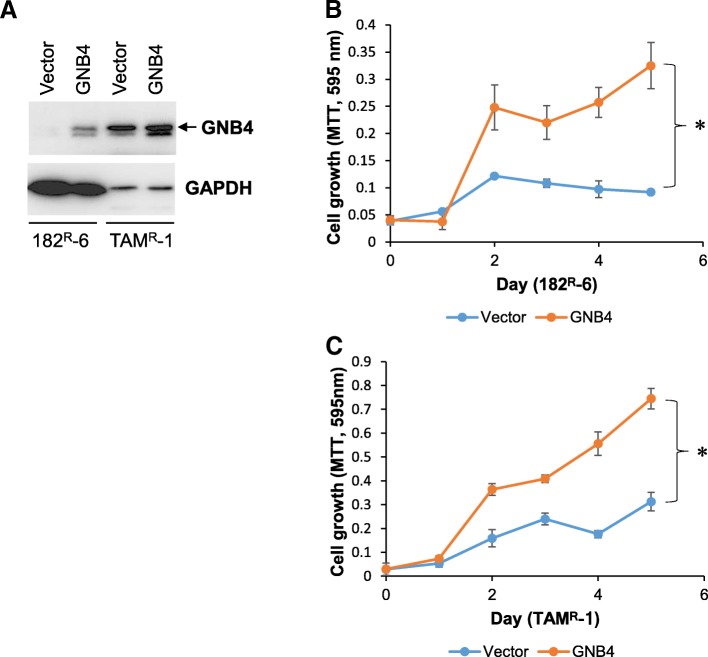
The ectopic expression of GNB4 enhances the proliferation of antiestrogen-resistant breast cancer cells. **a**, 182^R^-6 and TAM^R^-1 cells were transfected with either pEGFP-GNB4 or pEGFP-C1; after G418 selection, whole cellular lysate prepared from the positive cells was subjected to Western blot analysis using an antibody against GNB4. **b** and **c**, MTT assay was performed using 182^R^-6 (**b**) and TAM^R^-1 (**c**) cells stably expressing GNB4 or GFP as described in “Methods”. Asterisk indicates *p* < 0.05

Since GNB4 was downregulated in antiestrogen-resistant breast cancer cells, we hypothesized that GNB4 may function as an “antidrug-resistant” gene that may restore the sensitivity of resistant cell lines to either fulvestrant or tamoxifen. To test our hypothesis by determining the role of GNB4 in the development of acquired fulvestrant and tamoxifen resistance in breast cancer, we generated GNB4 stable-expressing 182^R^-6 and TAM^R^-1 cell lines (Fig. 3a). Surprisingly, the MTT assay showed that the ectopic expression of GNB4 further enhanced drug-resistant features of these cells by significantly promoting their proliferation (*p* < 0.05, Fig. 3b and c), even though they were under treatment with either fulvestrant or tamoxifen. Interestingly, in the fulvestrant- and tamoxifen-free medium growth conditions, GNB4 overexpression had no effect on 182^R^-6 cell growth (Additional file 1: Figure S1A), but suppressed TAM^R^-1 cell proliferation (Additional file 1: Figure S1B). Our results suggest that GNB4 is a drug-resistant gene in fulvestrant- and tamoxifen-resistant breast cancer cells.

### Ectopic expression of GNB4 altered cell cycle and apoptosis of antiestrogen-resistant breast cancer cells

**Fig. 4:**
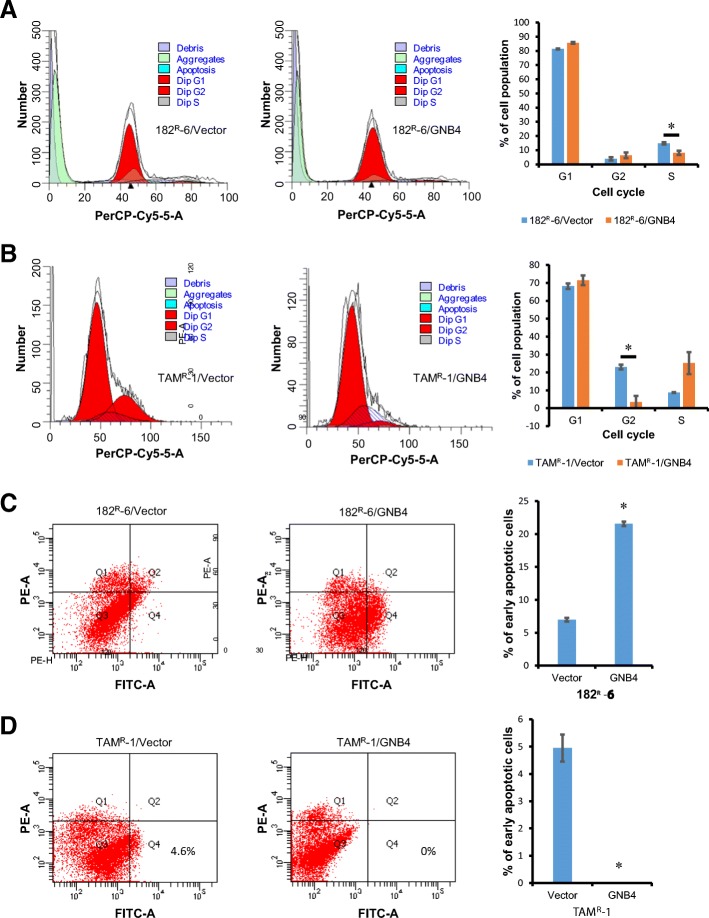
The ectopic expression of GNB4 causes alterations in cell cycle and apoptosis. **a** and **b**, 182^R^-6 and TAM^R^-1 cells stably expressing GNB4 or GFP grown to 90% confluency were subjected to cell cycle analysis using a GFP-Certified Nuclear-ID Red Cell Cycle Analysis Kit according to the manufacturer’s instructions. **c** and **d**, 182^R^-6 and TAM^R^-1 cells stably expressing GNB4 or GFP grown to 90% confluency were subjected to apoptosis analysis using an Annexin V-Cy3 Apoptosis Kit Plus according to the manufacturer’s instructions. Asterisk indicates *p* < 0.05

We next looked at the potential role of GNB4 in controlling the cell cycle and apoptosis of antiestrogen-resistant breast cancer cells. The cell-cycle analysis indicated that the ectopic expression of GNB4 shortened S-phase in 182^R^-6 cells and G2 in TAM^R^-1 cells (Fig. 4a and b). The apoptosis analysis showed that the enforced expression of GNB4 induced apoptosis of the 182^R^-6 cells, whereas it completely attenuated the induction of apoptosis in the TAM^R^-1 cells (Fig. 4c and d).

**Fig. 5:**
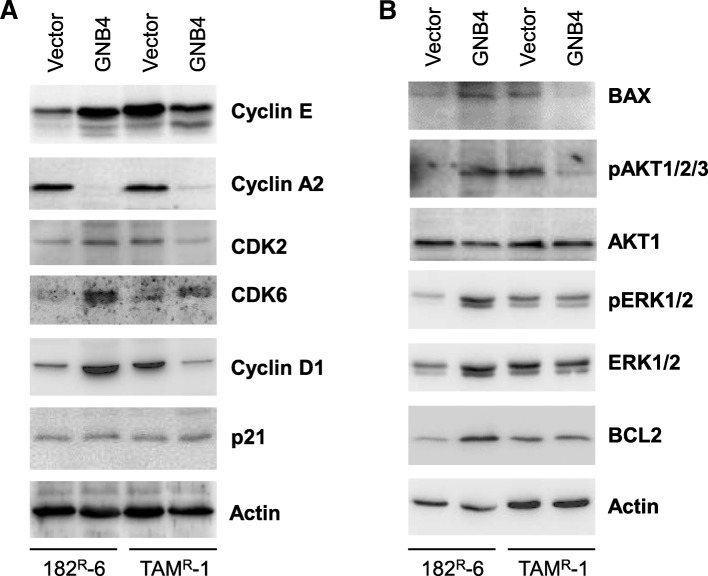
Changes of cell cycle and apoptosis regulators in response to enforced GNB4 expression. **a** and **b**, Whole cellular lysate prepared from 182^R^-6 and TAM^R^-1 cells stably expressing GNB4 or GFP was subjected to Western blot analysis using the indicated antibodies

To understand the mechanism underlying the GNB4-mediated alterations in proliferation, cell cycle, and apoptosis, we determined the expression of cell cycle and apoptosis regulators in 182^R^-6 and TAM^R^-1 cells in response to a GNB4 expression. The Western blot analysis showed that cyclin A2 was downregulated, while CDK6 was upregulated in 182^R^-6 and TAM^R^-1 cells in response to the ectopic expression of GNB4 (Fig. 5a). Interestingly, GNB4 caused an induction in genes, including those of cyclin D1 and E, CDK2, BAX, and phosphorylated Akt1/2/3 in 182^R^-6 cells, whereas it attenuated the expression of these gene in TAM^R^-1 cells (Fig. 5a and b). Although BCL2 and phosphorylated ERK1/2 were elevated in 182^R^-6 by GNB4, they had no effect in TAM^R^-1 cells (Fig. 5b). Additionally, GNB4 had no effect on p21 expression in both cell lines (Fig. 5a).

### Knockdown of GNB4 with siRNA suppressed proliferation and induced apoptosis and cell cycle arrest of antiestrogen-resistant breast cancer cells

**Fig. 6:**
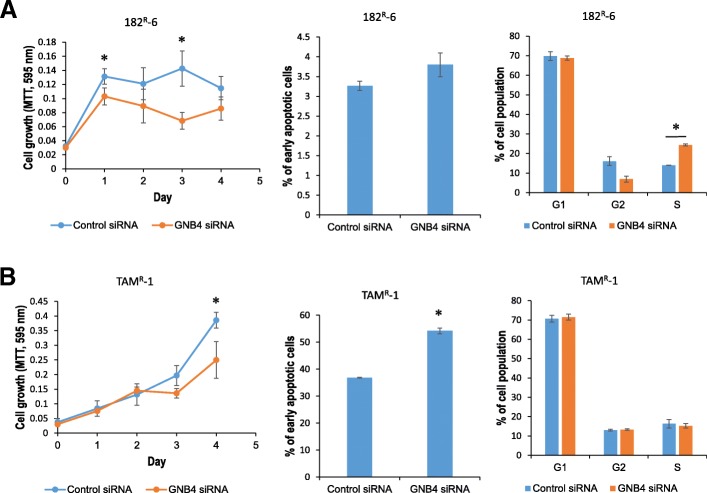
Knockdown of GNB4 inhibits proliferation and induces cell cycle arrest and apoptosis. **a** and **b**, 182^R^-6 and TAM^R^-1 cells grown to 80% confluency were transiently transfected with either 30 nM GNB4 siRNA or 30 nM negative control siRNA; 24 h after transfection, the cells were replated in 96-well plate; the MTT assay was performed as described in “Methods”; 72 h (TAM^R^-1) or 96 h (182^R^-6) after transfection, the cells were harvested for cell cycle and apoptosis analyses. Asterisk indicates *p* < 0.05

To further confirm the role of GNB4 in the control of cell proliferation, cell cycle and apoptosis in antiestrogen resistant 182^R^-6 and TAM^R^-1 cells, we then knocked down GNB4 using siRNA and measured the effect on cell proliferation, cell cycle and apoptosis. Western blot analysis showed that 72 h after transfection, the expression of GNB4 was attenuated in TAM^R^-1 cell line (Additional file 2: Figure S2), while had no effect in 182^R^-6 cell line. However, at 96 h after transfection, the expression of GNB4 was also reduced in 182^R^-6 cell line by GNB4 siRNA (Additional file 3: Figure S4). As expected, knockdown of GNB4 suppressed proliferation of 182^R^-6 and TAM^R^-1 cells in response to either fulvestrant or tamoxifen (Fig. 6a and b). Interestingly, Knockdown of GNB4 induced significantly apoptosis in TAM^R^-1 cells (Fig. 6b, middle panel), while had no effect on that in 182^R^-6 cells (Fig. 6a, middle panel). Furthermore, knockdown of GNB4 induced S-phase arrest in 182^R^-6 cells (Fig. 6a, right panel), whereas had no effect on that in TAM^R^-1 cells (Fig. 6b, right panel). Moreover, knockdown of GNB4 also inhibited proliferation of the parental S05 cells in the absence of antiestrogen drugs (Additional file 4: Figure S3), in addition to a role in drug resistance, may also implicating a role in the development of breast cancer.

## Discussion

Although the well-established and effective endocrine therapy has provided millions of women with ER+ breast cancer with targeted treatment option [20, 23], most patients with metastatic disease would unfortunately and inevitably develop resistance to the drugs [28, 29], which has become a major clinical challenge in the treatment of this disease. As demonstrated, the reactivation (reestablishment) of ER and growth-factor signaling through crosstalk has been proposed as a primary mechanism for the development of antiestrogen resistance. A central role of upregulated EGFR/ErbB and the sustained activation of the EGFR/ErbB/ERK signaling pathway has been strongly indicated in the maintenance of antiestrogen-resistant breast cancer cell growth [30–32]. Although ERα is downregulated in tamoxifen-resistant breast cancer cell lines, the receptor is highly activated (phosphorylated) [32]. Importantly, the activated ER has been shown to promote the expression of insulin-like growth factor II (IGF2) in tamoxifen-resistant cell lines, resulting in the reactivation of insulin-like growth factor 1 receptor (IGF1R) signaling that activates EGFR via c-src-dependent phosphorylation [33]. Interestingly, the activated IGF2/IGF1R signaling may therefore in turn contribute to the phosphorylation of ER in tamoxifen-resistant cell lines [33], hence forming a positive-feedback proliferation loop. In addition, changes in the sensitivity of antiestrogen-resistant breast cancer cells to estradiol (E2) may also play a pivotal role in the development of antiestrogen resistance. Several lines of evidence have demonstrated that long-term estrogen deprivation (LTED) enhances the sensitivity of breast cancer cells to low levels of E2 (~ 10,000-fold reduction) [34–36]. The biological effect induced by E2 hypersensitivity, such as proliferation, was also mediated by the activation (phosphorylation) of ER and ERK1/2 (extracellular signal-regulated kinase 1/2) signalings. More recently, several studies highlighted a key role of protein kinases in the development of antiestrogen resistance, such as tyrosine kinase FYN and aurora kinase A [37, 38]. Interestingly, the tamoxifen-resistant breast cancer cells may derive from cancer stem-like cells [39].

To our knowledge, this study has revealed, for the first time, that GNB4 was epigenetically silenced in antiestrogen-resistant breast cancer cells, and it has highlighted an important role of GNB4 in the growth of antiestrogen resistant breast cancer cells. Heterotrimeric G proteins which are composed of an alpha subunit, a beta subunit (e.g. GNB4), and a gamma subunit function as molecular switches that play a crucial role in signal transduction from a cell surface receptor to internal effectors in a G protein-coupled receptor (GPCR) pathway [40]. Although the GPCR signaling pathway has been extensively studied, very little is known about GNB4. However, since the GPCR pathway plays a pivotal role in many biologic and pathologic processes, including tumorigenesis, it may also reflect a role of GNB4 in these processes. Evidence has demonstrated that GNB-isoforms are essential for chemokine-induced GPCR downstream signaling [41]. GNB4 may be one of the key genes that cause Charcot-Marie-Tooth disease, a heterogeneous group of the inherited neuropathies [42, 43]. GNB4 has also been linked to cancer. Haplotypes of GNB4 intron-1 have been shown to be associated with the survival rate of patients with colorectal and urothelial bladder carcinomas [44, 45]. We showed here that GNB4 was downregulated in both fulvestrant- and tamoxifen-resistant breast cancer cell lines, and that this was attributed to DNMT3B-mediated DNA methylation, because knockdown of DNMT3B by siRNA significantly elevated the expression of GNB4 at both mRNA and protein levels (Fig. 2). GNB4 has been reported to be downregulated in progressive breast cancer due to the acquired tamoxifen resistance [46], which is consistent with our results. Importantly, we found that the ectopic expression of GNB4 remarkably promoted the proliferation of antiestrogen-resistant breast cancer cells in response to antiestrogen drugs (Fig. 3). We also noted that the enforced expression of GNB4 caused cell cycles G2 and S to undergo phase acceleration (Fig. 4a and b), which may contribute to the GNB4-mediated proliferation of antiestrogen-resistant cells (Fig. 3b and c). Interestingly, the ectopic expression of GNB4 completely abolished apoptosis in tamoxifen-resistant TAM^R^-1 cells, while it significantly induced apoptosis in fulvestrant-resistant 182^R^-6 cells (Fig. 4c and d). However, the dramatic effects of GNB4 on apoptosis in both cell lines may all contribute to GNB4-mediated cellular proliferation (Fig. 3b and c). Recently, several interesting studies have indicated that apoptotic cells could promote the proliferation of surrounding cells (apoptosis-induced proliferation) due to the mitogenic signals released by the apoptotic cells [47–49]. siRNA-mediated GNB4 knockdown, however, suppressed proliferation of 182^R^-6 and TAM^R^-1 cells in the presence of antiestrogen drugs, and induced S-phase arrest of 182^R^-6 cells and apoptosis of TAM^R^-1 cells (Fig. 6a and b), further validating a crucial role of GNB4 in the development of antiestrogen resistance of breast cancer cells. GNB4 siRNA had no effect on GNB4 expression in 182^R^-6 cells at 72 h after transfection (Additional file 2: Figure S2), may reflect a longer half-life time of GNB4 in this cell line, since in another independent experiment, GNB4 was noted to be downregulated at 96 h after transfection (Additional file 3: Figure S4A and B).

GNB4 is a key component of heterotrimeric G proteins, which play an essential role in the transduction of GPCR-mediated signaling. Because of the crucial role of GPCR in the activation of AKT (v-AKT murine thymoma viral oncogene homolog) and ERK1/2 pathways [50, 51], an increase in phosphorylated AKT and/or phosphorylated ERK1/2 was expected to be seen in antiestrogen-resistant cells in response to the ectopic GNB4 expression. As expected, the phosphorylated AKT and phosphorylated ERK1/2 were elevated in 182^R^-6 cells in response to the GNB4 expression (Fig. 5b). This may contribute to GNB4-mediated cellular proliferation (Fig. 3b). However, the enforced expression of GNB4 caused a reduction in the phosphorylated AKT and had no effect on the phosphorylated ERK1/2 in TAM^R^-1 cells (Fig. 5b). The mechanism involved is unclear. It may be interesting to look at the crosstalk with other pathways, such as ER. Importantly, the GNB4-induced upregulation of BAX in 182^R^-6 cells and the downregulation in TAM^R^-1 cells may contribute to GNB4’s effect on apoptosis in these cells (Fig. 5b and Fig. 4c and d). We also noted that GNB4 caused an induction in BCL2 in 182^R^-6 cells, while it had no effect in TAM^R^-1 cells (Fig. 5b), implicating that the functional effect of GNB4 on apoptosis in 182^R^-6 cells may be due to the balance between proapoptotic (BAX) and antiapoptotic (BCL2) proteins.

Cyclins and cyclin-dependent kinases control cell-cycle progression and transitions. Cyclin A interacts with cyclin-dependent kinase 2 (CDK2) or CDK1 to form a complex that governs the S phase of the cell cycle [52]. Cyclin D interacts with CDK4 or CDK6 to form a complex that controls the G1 phase of the cell cycle [49]. However, in a complex with CDK2, cyclin E controls the S-phase progression and G1-S transition [52, 53]. The results we presented here showed that cyclin E and CDK2 were upregulated in 182^R^-6 cells in response to GNB4 (Fig. 5a), which may contribute to the shortened S phase (Fig. 4a). The ectopic GNB4, however, attenuated the expression of CDK2 and cyclin A and E in TAM^R^-1 cells (Fig. 5a), which may contribute to the S-phase arrest (Fig. 4a). Although the ectopic GNB4 caused a profound induction in both cyclin D1 and CDK6 in 182^R^-6 cells (Fig. 5a), this induction had no effect on the G1 phase of the cell cycle (Fig. 4a). The GNB4-induced upregulation of CDK6 and downregulation of cyclin D1 had no effect on the G1 phase of TAM^R^-1 cells (Fig. 5a and Fig. 4b). We also noted that the ectopic GNB4 had no effect on the expression of p21, an inhibitor of cyclin D/CDK6 and cyclin E/CDK2 complexes [54]. However, the mechanism underlying the shortened GNB4-induced G2 phase of the cell cycle in TAM^R^-1 cells is still unclear. Although we did not measure the expression of CDC25 and speedy/ringo C in these cells, it has been demonstrated that these two molecules play an important role in controlling G2-phase progression [55, 56].

## Conclusion

In summary, although the observed reduced level of GNB4 in the two antiestrogen resistant cell lines is not the underlying cause of antiestrogen resistance, GNB4 is important for growth of both antiestrogen resistant and antiestrogen sensitive breast cancer cells and thereby a target for treatment of breast cancer.

## Additional files

Additional file 1: **Figure S1.** Effect of GNB4 overexpression on cell growth of 182^R^-6 and TAM^R^-1 cell lines (no drug treatment). A and B, MTT assay was performed using 182^R^-6 (a) and TAM^R^-1 (b) cells stably expressing GFP or GNB4 as described in “Methods”, using fulvestrant- and tamoxifen-free medium. Asterisk indicates *p* < 0.05. (PPTX 42 kb)

Additional file 2: **Figure S2.** Knockdown of GNB4 in TAM^R^-1 and 182^R^-6 cells using siRNA. 182^R^-6 and TAM^R^-1 cells grown to 80% confluency were transiently transfected with either 30 nM GNB4 siRNA or 30 nM negative control siRNA; at 72 h after transfection, whole cellular lysates were prepared and subjected to Western blot analysis using antibody against GNB4. (PPTX 508 kb)

Additional file 3: **Figure S4.** siRNA-mediated knockdown of GNB4 in TAM^R^-1 and 182^R^-6 cells. A, 182^R^-6 and TAM^R^-1 cells grown to 80% confluency were transiently transfected with either 30 nM GNB4 siRNA or 40 nM negative control siRNA; At 72 and 96 h after transfection, whole cellular lysates were prepared and subjected to Western blot analysis using antibody against GNB4. B, a relative densitometry (GNB4/Actin) was performed to further validate the GNB4 expression in 182^R^-6 cell line 96 h after transfection, using ImageJ software. Asterisk indicates *p* < 0.05. (PPTX 49 kb)

Additional file 4: **Figure S3.** Knockdown of GNB4 using siRNA suppresses proliferation of parental S05 cells. S05 cells grown to 80% confluency were transiently transfected with either 30 nM GNB4 siRNA or 30 nM negative control siRNA; at 24 h after transfection, the cells were replated in 96-well plate, MTT assay was performed as described in “Methods”. Asterisk indicates *p* < 0.05. (PPTX 37 kb)

## Abbreviations

AKT1: V-AKT murine thymoma viral oncogene homolog 1
BAX: BCL2-associated X protein
BCL2: B-cell CLL/Lymphoma 2
CDK2: cyclin-dependent kinase 2
CDK6: cyclin-dependent kinase 6
DBD: DNA-binding domain
DNMT1: DNA methyltransferase 1
DNMT3A: DNA methyltransferase 3A
DNMT3B: DNA methyltransferase 3B
ER: Estrogen receptor
ERK1/2: Extracellular signal-regulated kinase 1/2
GAPDH: Glyceraldehyde-3-phosphate dehydrogenase gene
GNB4: Guanine nucleotide-binding protein beta-4
HMEC: Human mammary epithelial cells
LBD: Ligand-binding domain
MeCP2: Methyl-CpG-binding protein 2
MTT: The 3-(4,5-Dimethylthiazol-2-yl)-2,5-diphenyl tetrazolium bromide
NTD: N-terminal domain
qRT-PCR: Quantitative real-time RT-PCR
siRNA: Small interfering RNA
