# Supplementary figures and images for: A suppressive role of guanine nucleotide-binding protein subunit beta-4 inhibited by DNA methylation in the growth of anti-estrogen resistant breast cancer cells
Source: BMC Cancer. 2018 Aug 13;18:817. doi: 10.1186/s12885-018-4711-0 (PMC6090602; doi:10.1186/s12885-018-4711-0)

## Slide 1
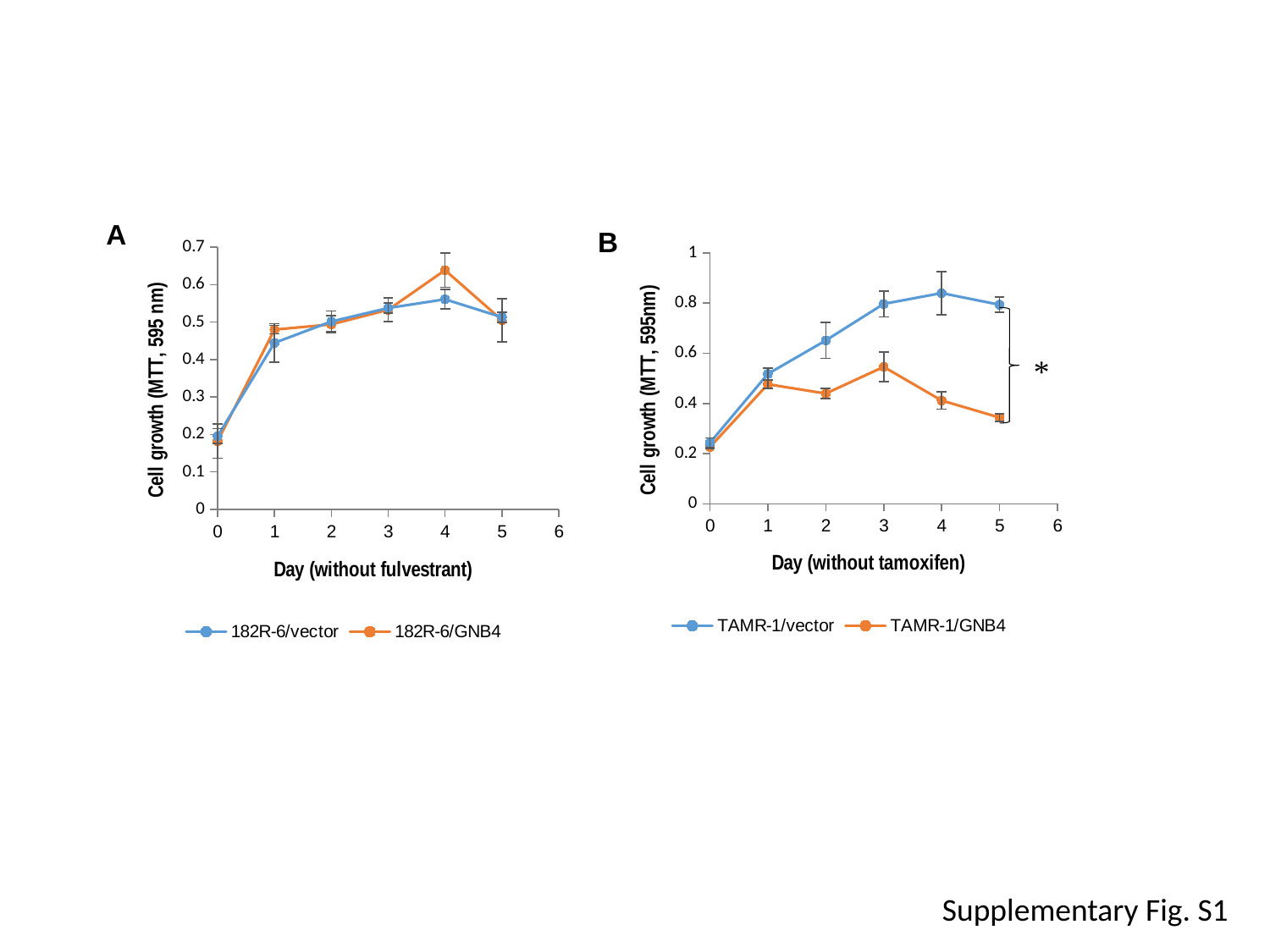

A
B
### Chart
| Category | 182R-6/vector | 182R-6/GNB4 |
|---|---|---|
### Chart
| Category | TAMR-1/vector | TAMR-1/GNB4 |
|---|---|---|
*
Supplementary Fig. S1

Supplement: Supplementary file 1 — Figure S1. Effect of GNB4 overexpression on cell growth of 182R-6 and TAMR-1 cell lines (no drug treatment). A and B, MTT assay was performed using 182R-6 (a) and TAMR-1 (b) cells stably expressing GFP or GNB4 as described in “Methods”, using fulvestrant- and tamoxifen-free medium. Asterisk indicates p < 0.05. (PPTX 42 kb) [file 12885_2018_4711_MOESM1_ESM.pptx]
