# Supplementary material for: A suppressive role of guanine nucleotide-binding protein subunit beta-4 inhibited by DNA methylation in the growth of anti-estrogen resistant breast cancer cells
Source: BMC Cancer. 2018 Aug 13;18:817. doi: 10.1186/s12885-018-4711-0 (PMC6090602; doi:10.1186/s12885-018-4711-0)
Supplement: Supplementary file 2 — Figure S2. Knockdown of GNB4 in TAMR-1 and 182R-6 cells using siRNA. 182R-6 and TAMR-1 cells grown to 80% confluency were transiently transfected with either 30 nM GNB4 siRNA or 30 nM negative control siRNA; at 72 h after transfection, whole cellular lysates were prepared and subjected to Western blot analysis using antibody against GNB4. (PPTX 508 kb) [file 12885_2018_4711_MOESM2_ESM.pptx]

## Slide 1
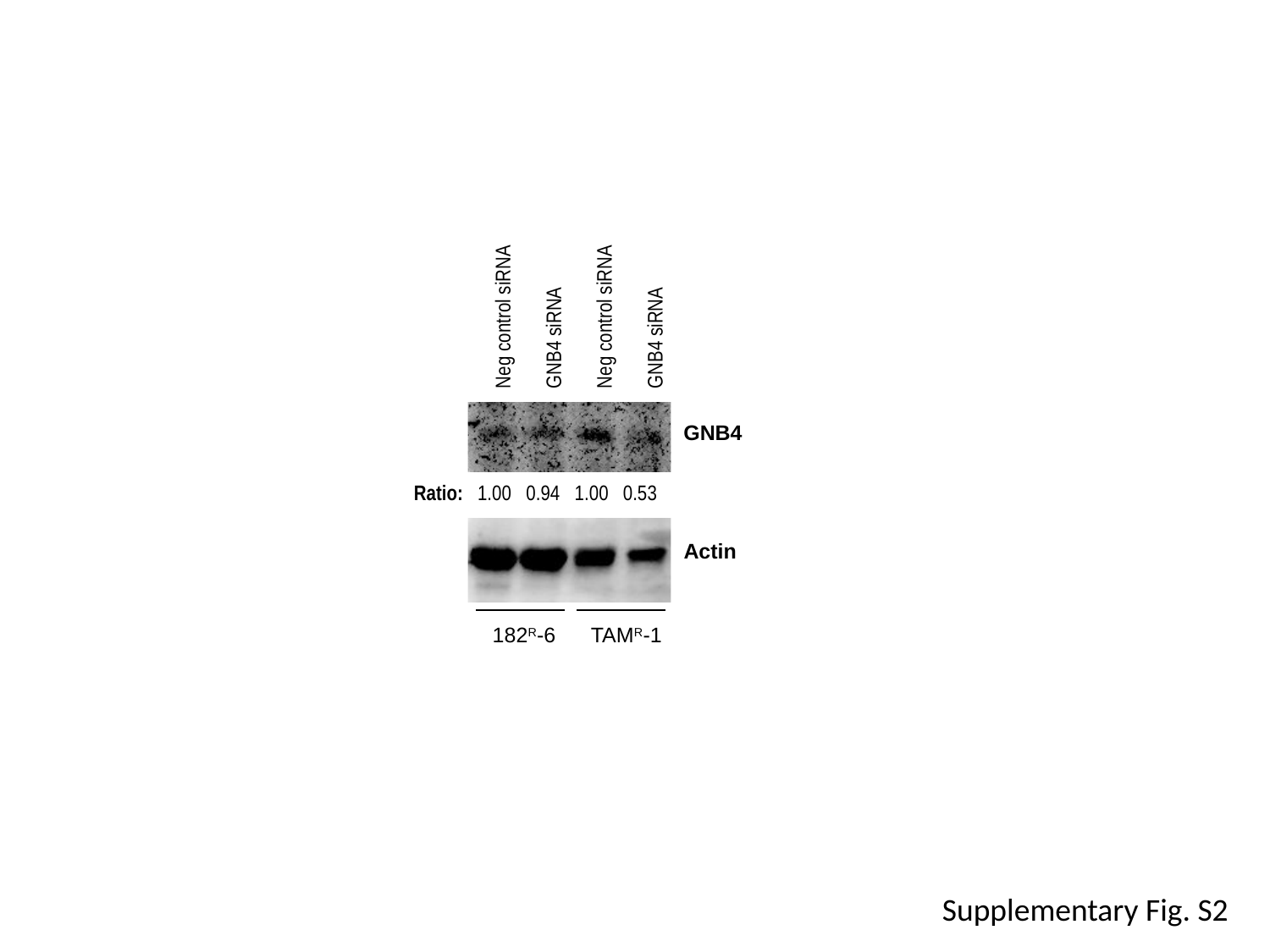

Neg control siRNA
GNB4 siRNA
Neg control siRNA
GNB4 siRNA
GNB4
Ratio: 1.00 0.94 1.00 0.53
Actin
182R-6 TAMR-1
Supplementary Fig. S2
