# Supplementary material for: A suppressive role of guanine nucleotide-binding protein subunit beta-4 inhibited by DNA methylation in the growth of anti-estrogen resistant breast cancer cells
Source: BMC Cancer. 2018 Aug 13;18:817. doi: 10.1186/s12885-018-4711-0 (PMC6090602; doi:10.1186/s12885-018-4711-0)
Supplement: Supplementary file 3 — Figure S4. siRNA-mediated knockdown of GNB4 in TAMR-1 and 182R-6 cells. A, 182R-6 and TAMR-1 cells grown to 80% confluency were transiently transfected with either 30 nM GNB4 siRNA or 40 nM negative control siRNA; At 72 and 96 h after transfection, whole cellular lysates were prepared and subjected to Western blot analysis using antibody against GNB4. B, a relative densitometry (GNB4/Actin) was performed to further validate the GNB4 expression in 182R-6 cell line 96 h after transfection, using ImageJ software. Asterisk indicates p < 0.05. (PPTX 49 kb) [file 12885_2018_4711_MOESM3_ESM.pptx]

## Slide 1
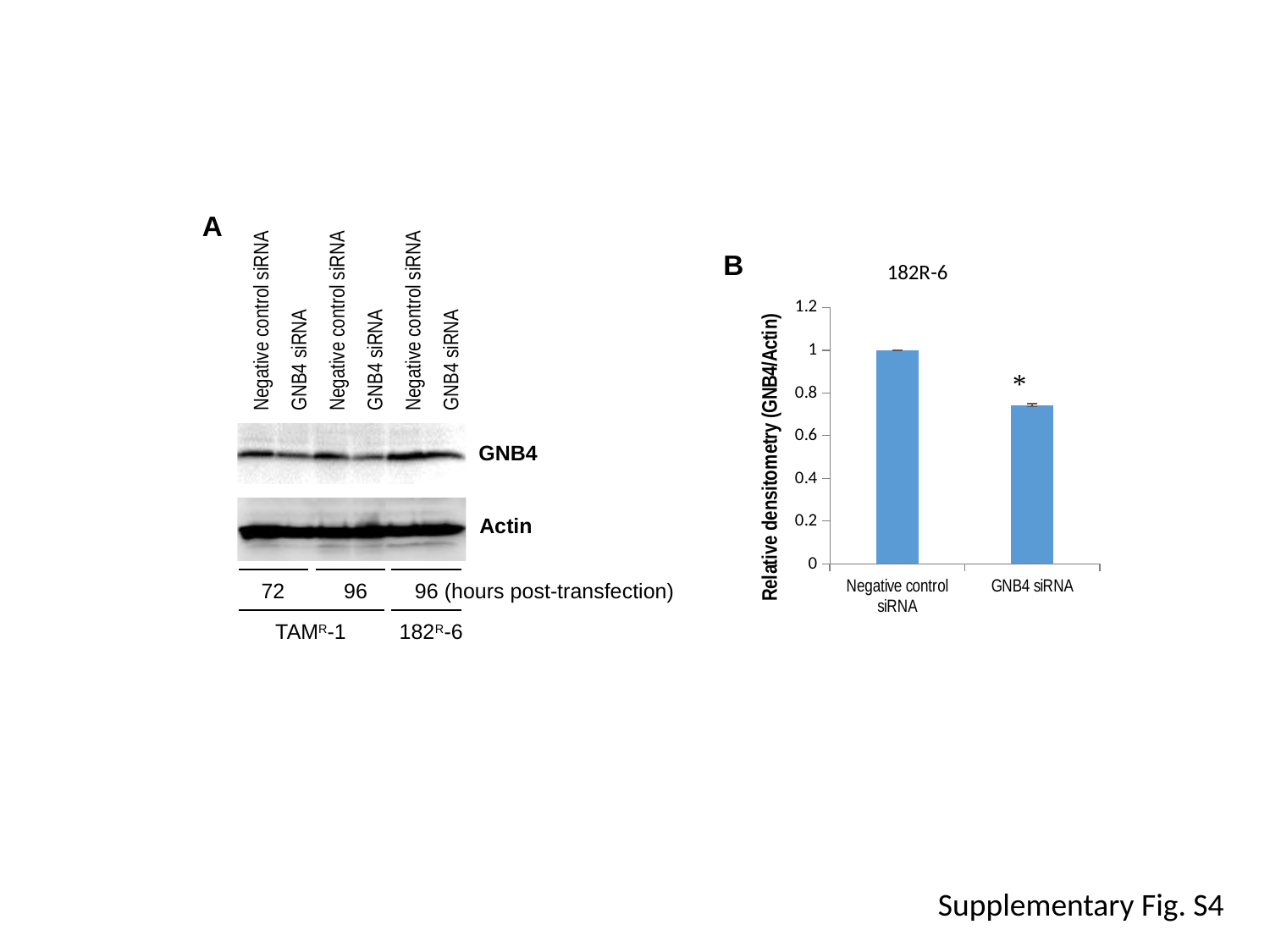

Negative control siRNA
GNB4 siRNA
Negative control siRNA
GNB4 siRNA
Negative control siRNA
GNB4 siRNA
A
B
### Chart: 182R-6
| Category | |
|---|---|
| Negative control siRNA | 1.0 |
| GNB4 siRNA | 0.7435768829358284 |*
GNB4
Actin
72 96 96 (hours post-transfection)
TAMR-1 182R-6
Supplementary Fig. S4
