# Supplementary material for: A suppressive role of guanine nucleotide-binding protein subunit beta-4 inhibited by DNA methylation in the growth of anti-estrogen resistant breast cancer cells
Source: BMC Cancer. 2018 Aug 13;18:817. doi: 10.1186/s12885-018-4711-0 (PMC6090602; doi:10.1186/s12885-018-4711-0)
Supplement: Supplementary file 4 — Figure S3. Knockdown of GNB4 using siRNA suppresses proliferation of parental S05 cells. S05 cells grown to 80% confluency were transiently transfected with either 30 nM GNB4 siRNA or 30 nM negative control siRNA; at 24 h after transfection, the cells were replated in 96-well plate, MTT assay was performed as described in “Methods”. Asterisk indicates p < 0.05. (PPTX 37 kb) [file 12885_2018_4711_MOESM4_ESM.pptx]

## Slide 1
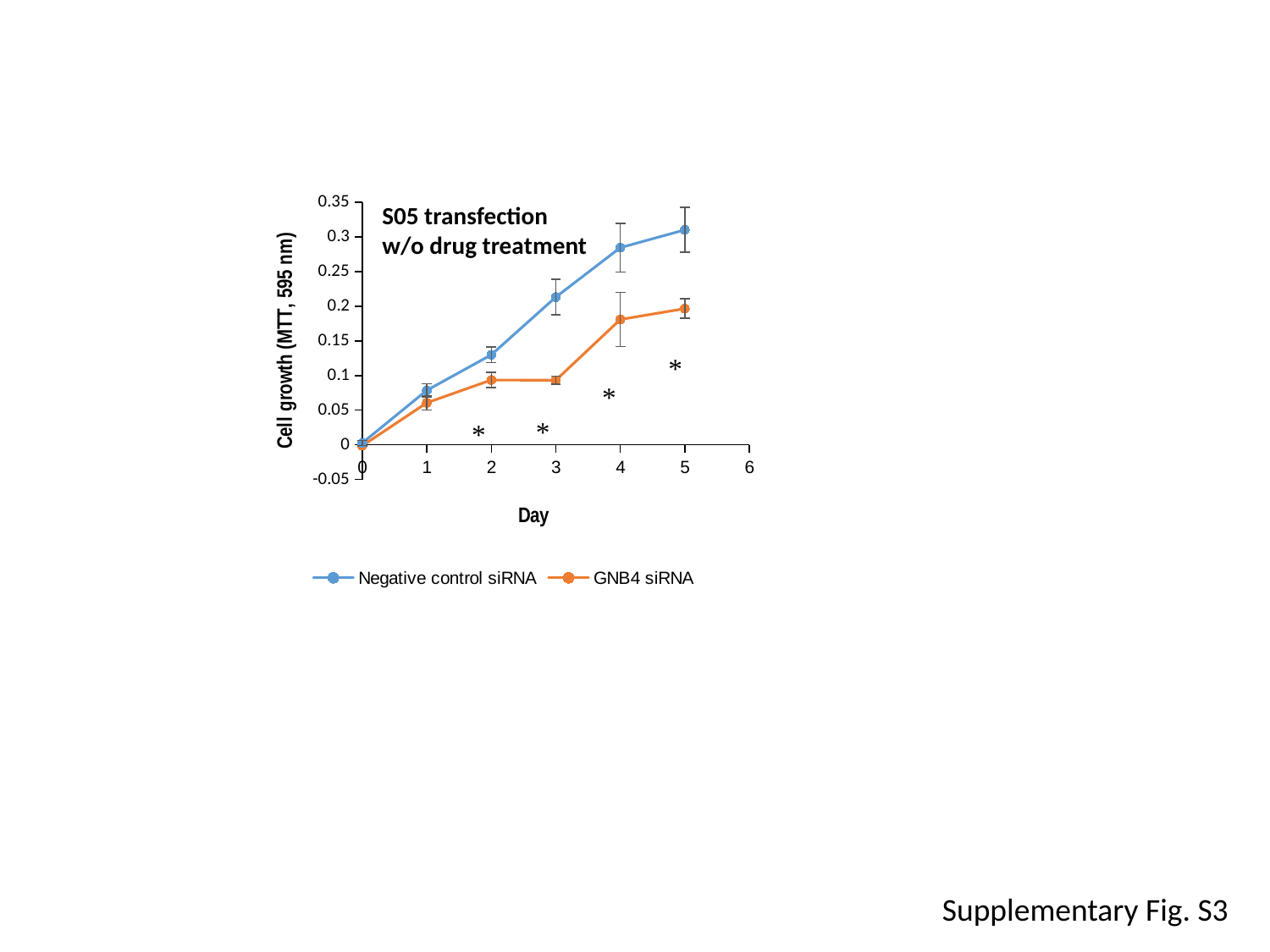

### Chart
| Category | Negative control siRNA | GNB4 siRNA |
|---|---|---|S05 transfection
w/o drug treatment
*
*
*
*
Supplementary Fig. S3
